# Nature Has No Elementary Particles and Makes No Measurements or Predictions: Quantum Measurement and Quantum Theory, from Bohr to Bell and from Bell to Bohr

**DOI:** 10.3390/e23091197

**Published:** 2021-09-11

**Authors:** Arkady Plotnitsky

**Affiliations:** Literature, Theory and Cultural Studies Program, Philosophy and Literature Program, Purdue University, West Lafayette, IN 47907, USA; plotnits@purdue.edu

**Keywords:** quantum measurement, quantum objects, quantum fields, reality without realism

## Abstract

This article reconsiders the concept of physical reality in quantum theory and the concept of quantum measurement, following Bohr, whose analysis of quantum measurement led him to his concept of a (quantum) “phenomenon,” referring to “the observations obtained under the specified circumstances,” in the interaction between quantum objects and measuring instruments. This situation makes the terms “observation” and “measurement,” as conventionally understood, inapplicable. These terms are remnants of classical physics or still earlier history, from which classical physics inherited it. As defined here, a quantum measurement does not measure any preexisting property of the ultimate constitution of the reality responsible for quantum phenomena. An act of measurement establishes a quantum phenomenon by an interaction between the instrument and the quantum object or in the present view the ultimate constitution of the reality responsible for quantum phenomena and, at the time of measurement, also quantum objects. In the view advanced in this article, in contrast to that of Bohr, quantum objects, such as electrons or photons, are assumed to exist only at the time of measurement and not independently, a view that redefines the concept of quantum object as well. This redefinition becomes especially important in high-energy quantum regimes and quantum field theory and allows this article to define a new concept of quantum field. The article also considers, now following Bohr, the quantum measurement as the entanglement between quantum objects and measurement instruments. The argument of the article is grounded in the concept “reality without realism” (RWR), as underlying quantum measurement thus understood, and the view, the RWR view, of quantum theory defined by this concept. The RWR view places a stratum of physical reality thus designated, here the reality ultimately responsible for quantum phenomena, beyond representation or knowledge, or even conception, and defines the corresponding set of interpretations quantum mechanics or quantum field theory, such as the one assumed in this article, in which, again, not only quantum phenomena but also quantum objects are (idealizations) defined by measurement. As such, the article also offers a broadly conceived response to J. Bell’s argument “against ‘measurement’”.

*Even in a low-brow practical account, I think it would be good to replace the word ‘measurement’, in the formulation, by the word ‘experiment’. For the latter word is altogether less misleading. However, the idea that quantum mechanics, our most fundamental physical theory, is exclusively even about the results of experiments would remain disappointing.*

—*John Bell, “Against ‘measurement’”* [1] (pp. 216–217)

## 1. Introduction

This article reconsiders the concept of physical reality in quantum theory and the concept of quantum measurement, following Bohr, whose analysis of quantum measurement led him to his concept of “phenomena,” as applicable in quantum mechanics (QM) and quantum field theory (QFT). This concept is defined “by the application of the word *phenomenon* exclusively to refer to the observations obtained under the specified circumstances”arising in the interactions between quantum objects and measuring instruments, which interactions are, according to Bohr, irreducible in the constitution of quantum phenomena, as against the phenomena considered of classical physics or relativity, where the role of measuring instruments can be disregarded or compensated for [2] (v. 2, p. 64). It is this situation that is responsible for the concept of quantum measurement proposed in this article. This concept makes the terms “observation” and “measurement,” as conventionally understood, inapplicable in considering quantum phenomena. These terms are remnants of classical physics and still earlier history, from which classical physics inherited them, beginning with ancient Greek thinking and the rise of geometry (geo-*metry*) there. As understood here, a quantum measurement does not measure any preexisting property of the ultimate constitution of the reality responsible for quantum phenomena. Hence, the concept of observation requires a different understanding as well. An act of observation in quantum physics establishes quantum phenomena by an interaction between the instrument and the quantum object or in the present view the ultimate constitution of the reality responsible for quantum phenomena and, at the time of measurement, also quantum objects. In this sense, one might say that the so-called “quantum measurement problem” does not exist because there is no measurement as conventionally understood. There are only quantum interactions between the ultimate constitution of nature, the constitution assumed to be quantum and our observational technology and the classical outcomes of these interactions.

I refer to the ultimate constitution of nature because, in the view adopted in this article, quantum objects are *idealized* as entities existing only at the time of measurement and not independently. This view, accordingly, also redefines the concept of quantum object, including that of an elementary particle. Hence, as stated in my title, the present interpretation of quantum phenomena no longer assumes that the concept of an elementary particle could be attributed to nature itself. What is observed as the data or information can then be measured classically, in classical bits, just as one measures what is observed in classical physics, where, however, what is so measured could be associated with the object itself considered, for all practical purposes. As far as the observed data or information is concerned, a quantum measurement is not a measurement of anything but a number or bit generator, akin to a quantum computer created by our interaction with nature. With this concept of measurement in hand, the article will also discuss, following Bohr, a quantum measurement as the entanglement between the quantum object and the measurement instrument. Finally, it will consider the question of measurement in high-energy quantum physics and QFT, where the article’s redefinition of the concept of a quantum object, as only applicable at the time of measurement, becomes especially important, also allowing the article to define a new concept of quantum field.

As such, the article offers a broadly conceived response to J. Bell’s argument “against ‘measurement’,” in his article under this title [1]. I speak of a broadly conceived response because my aim is not an analysis or criticism of Bell’s argument itself, beneficially discussed by commentators (e.g., [3] and references there). The present article suggests a view that does not appear to be considered by Bell in this article (or elsewhere), a view that he was unlikely to welcome any more than that of Bohr, for which Bell had little sympathy, including in “Against ‘measurement’.” The present article is a response to Bell because, against Bell’s own grain, Bell’s criticism of QM and ‘measurement’ (his quotation marks must be kept in mind, as indicating that he does not merely reject the idea of measurement) helps the present argument concerning quantum measurement. Consider Bell’s statement used as my epigraph:

*Even in a low-brow practical account, I think it would be good to replace the word ‘measurement’, in the formulation, by the word ‘experiment’. For the latter work is altogether less misleading. However, the idea that quantum mechanics, our most fundamental physical theory, is exclusively even about the results of experiments would remain disappointing.*

[1] (pp. 216–217)

As should be clear from the introductory comments above and as this article will argue, Bell is right in proposing to replace “measurement” with “experiment.” On the other hand, this assessment is read here against Bell’s own grain, by following Bohr’s concept of phenomena referring to “the observations obtained under the specified circumstances,” and (Bell might not have especially objected to Bohr’s sentence as such) especially by adopting and even extending its grounding premises and implications, as this article does. Thus, the present view assumes, first, that the ultimate constitution of the reality responsible for quantum phenomena is beyond representation or even conception, and second, that the concept of quantum objects is only applicable at the time of such observations, rather than independently. The first assumption would, as suggested by this statement and Bell’s overall realist view, be seen by Bell as “disappointing” at best, and the second does not appears to have been contemplated by him at all. It would, however, be difficult to think that Bell would have seen this second assumption as any less disappointing than the first. Indeed, the second assumption does not appear to have been contemplated by Bohr either, but in contrast to that of Bell, it is in affinity with and may even be seen as implied by Bohr’s argument. It also follows, however, that, Bohr’s (or the present) view would not correspond to the view (which Bell clearly associates with Bohr) “that quantum mechanics, our most fundamental physical theory, is *exclusively even* about the results of experiments” (emphasis added). *Bohr’s or the present view is about the ultimate nature of the reality responsible for quantum phenomena*. As, however, Bohr said in responding to A. Einstein’s concerns, similar to those of Bell: “In quantum mechanics, we are not dealing with an arbitrary renunciation of a more detailed analysis of atomic [quantum] phenomena but with a recognition that such an analysis is *in principle* excluded,” referring by phenomena to his sense of phenomena as “the observations obtained under the specified circumstances” and thus to the outcomes of quantum experiments [2] (pp. 62, 64). This argument was rejected, rather than counterargued, by Einstein as “so very contrary to [his] scientific instinct” [2] (pp. 61–62), an instinct shared by Bell. Nevertheless, just as that of Einstein, which helped Bohr to develop his argument, Bell’s critique helps us explore new trajectories of thinking about quantum phenomena, including measurement, and QM or QFT.

The argument of the article is grounded in the concept “reality without realism” (RWR), suggested by Bohr’s statement just cited and the view, the RWR view, defined by this concept, of quantum theory, specifically QM and QFT in their standard versions, referring by QFT, unless qualified, to high-energy, relativistic versions of it. (Alternative theories of quantum phenomena, such Bohmian mechanics, will only be mentioned in passing.) The RWR view places a stratum of physical reality thus designated, here the one ultimately responsible for quantum phenomena, beyond representation or knowledge, which I define as the *weak* RWR view, or even conception, which I define as the *strong* RWR view (assumed in this article), and thus defines the corresponding set of interpretations, weak and strong, of quantum phenomena and QM or QFT. The concept of reality without realism presupposes general concepts of reality and existence, discussed in detail below. Briefly, by reality, I understand that which is assumed to exist without making any claims concerning the *character* of this existence. Such claims define realism, which, in most understandings of the term, assumes the possibility of forming an (usually idealized) representation by means of one or another theory or at least a conception of the reality responsible for the phenomena considered. By contrast, the absence of such a claim allows one to place a given reality or part of it beyond representation or knowledge, or conception, in accord with the RW view.

The concept of reality without realism and the RWR view were introduced by this author previously (e.g., [4,5,6,7,8]). They will, however, be given a more radical form in this article by virtue of redefining the concept of quantum objects as only applicable at the time of measurement, which further stratifies the concept of quantum measurement adopted by this article, in contrast to Bohr’s interpretation. In general, RWR-type interpretations may be different within each set, weak or strong, and Bohr’s interpretation, in its ultimate version (developed by him in the late 1930s, as a revision of earlier versions), and the one adopted here are different, although both are of the strong RWR-type and the present one follows that of Bohr in several key aspects. In particular, Bohr was, again, the first to ground his interpretation, in all of its versions, in the irreducible role of measurement instruments in the constitution of quantum phenomena and, in its ultimate version, in the RWR-type concept of reality as applied to the ultimate constitution of the reality responsible for quantum phenomena, although he did not speak in terms of reality without realism. This constitution is, in Bohr’s interpretation, associated with quantum objects, as is common. The present interpretation adopts a more stratified view, which redefines the concept of quantum objects, a view proposed by this author earlier in the context of QFT [9]. This view is as follows.

While what is considered as a quantum object in a given experiment is made possible by the ultimate constitution of the reality responsible for quantum phenomena in its interaction with the measuring instruments used, and while these two strata of reality are equally beyond conception, they are not the same forms of idealization. The ultimate constitution of the reality responsible for quantum phenomena is an idealization assumed to exist independently of our interactions with it and thus independently of observation. On the other hand, the reality idealized as quantum objects is, while still of the RWR-type, assumed to exist only *at the time observation*. It follows that, in this interpretation, there are no quantum objects, such as electrons, photons, or quarks existing independently in nature apart from our interaction with it by means of our observational technology. Hence, one cannot speak of the behavior of quantum objects as independent of observation either. One can only consider this behavior as part of observations or measurement, again, understanding by measurement the construction of quantum phenomena by means of measuring instruments capable to interaction with the ultimate, RWR-type, constitution of the reality responsible for quantum phenomena. This view, on this point following Bohr, makes it impossible to separate, extract, quantum objects from quantum phenomena observed in measuring instruments. Bohr spoke in this connection of the indivisibility or wholeness of quantum phenomena, which makes it impossible to consider the behavior of quantum objects independently, even if one assumes, that they exist independently (e.g., [2] (v. 2, pp. 61, 72)). Quantum phenomena themselves, as defined by effects observed in measuring instruments, allow for a representational and thus realist treatment, as do, in the first place, measuring instruments, or more accurately, their observable parts. They also have quantum strata through which they interact with quantum objects, as considered below. In Bohr’s interpretation and, following it, the present interpretation, both the observable parts of measuring instruments and, hence, quantum phenomena are treated by means of classical physics. The *quantum* character of quantum phenomena is defined by the particular configurations of their classically observed features. Eventually, Bohr adopted the term “phenomenon,” when used in quantum physics, as referring to what is observed or, more precisely, what *has already been* observed, in specified setups, in measuring instruments, as effects of their interaction with quantum objects [2] (v. 2, p. 64). It is, as will be seen, crucial that Bohr’s concept refers to what has already been observed rather than only predicted.

The remainder of this article proceeds as follows. The next section outlines its key concepts. Section 3 discusses the concept of quantum measurement. Section 4 discusses the quantum measurement as an entanglement. Section 5 considers measurement, quantum objects, and elementary particles in QFT.

## 2. An Outline of Concepts

This section outlines the key concepts considered in this article, both those specifically grounding my argument, such as that of reality without realism or quantum object, and more commonly used concepts, such as reality, causality, or determinism, in their definitions adopted here, because they can be defined otherwise. I begin with the concepts of reality, realism, and reality without realism. As indicated in the Introduction, the concept of reality without realism, RWR, is grounded in more general concepts of reality and existence, assumed here to be primitive concepts and not given analytical definitions. These concepts are, however, in accord with most, even if not all (which would be impossible), available concepts of reality and existence in realism and nonrealism alike. By reality I refer to that which is assumed to exist, without making any claims concerning the *character* of this existence. The absence of such claims, which define realism, allows one to place this character beyond representation or even conception, as, in the case of the ultimate nature of the reality responsible for quantum phenomena, in RWR-type interpretations. I understand existence as a capacity to have effects on the world with which we interact. The very assumption that something is real, including of the RWR-type, is made on the basis of such effects. Following L. Wittgenstein, I understand “the world” as “everything that is the case,” in particular (but not exclusively) the world of events [10] (p. 1). This also includes entities and events, including mental (for example, mathematical), of the human world. Quantum events are observed as phenomena defined by measuring instruments or their equivalents in nature. On the other hand, while unobservable, the ultimate constitution of the reality responsible for quantum phenomena in the RWR view is assumed to be “the case” as well and is, thus, part of the world. It never appears as such and hence is not an event, but it manifests itself in events, from which its existence is inferred.

A given theory or interpretation might assume different levels and different types of *idealizations* of reality, some allowing for a representation or conception and others not. (By “idealization” I refer a workable conception of something or, in the strong RWR view, a lack of such a conception, thus, possibly, something different from what it idealizes, rather than, as some do, to any form of approximation of something.) As stated in the Introduction, the present interpretation of quantum phenomena and QM or QFT assumes three idealizations within its overall idealization scheme. The behavior of the observable parts of measuring instruments, defining quantum phenomena, is idealized as representable. By contrast, the RWR-type reality ultimately responsible for these phenomena is idealized as that which cannot be represented or even conceived of. The third idealization is that of quantum objects. The reason for assuming the latter idealization is as follows. On the one hand, in contrast to classical physics or relativity, in quantum physics, in each experimental arrangement one must, as Bohr argued, always discriminate “between those parts of the physical system considered which are to be treated as measuring instruments and those which constitute the objects under investigation” [11] (p. 701). One the other hand, the difference between these two parts is, in general, not uniquely defined, which is sometimes expressed as the arbitrariness of the “cut,” discussed in the next section. Accordingly, it is how we set up and interpret an experiment that defines what is the quantum object in this experiment and thus brings this object into existence, still as an RWR-type entity, in the present interpretation. Its quantum nature, including as an RWR-type entity, is defined by the ultimate, RWR-type, reality, which, by contrast, exist independently of any experiment.

Realist thinking is manifested in the corresponding realist theories (the terms like “ontic” and “ontological” are sometimes used as well), which are commonly representational in character. Such theories aim to represent the reality they consider, usually by mathematized models based on suitably idealizing this reality. It is possible to aim, including in quantum theory, for a strictly mathematical representation of this reality apart from physical concepts, at least as they are customarily understood, as in classical physics or relativity. It is also possible only to assume an independent architecture, structure, of the reality considered, without assuming that it is either (A) not possible to adequately represent this architecture by means of a physical theory, or (B) even to form a rigorously specified concept of this architecture, either at a given moment in history or even ever. Under (A), a theory that is merely predictive could be accepted for lack of a realist alternative, but usually with the hope that a representational theory will eventually be developed. This was Einstein’s attitude toward QM, or QFT, which he expected to be eventually replaced by a realist theory. What, then, grounds realism most fundamentally is the assumption that the ultimate constitution of reality possesses properties and the relationships between them, or, as in (ontic) structural realism [12], just a structure, in particular, a mathematical structure. This constitution may either be ideally represented and, hence, known or be unrepresented or unknown, or even unrepresentable or unknowable, but still conceivable, usually with a hope that it will be eventually represented and known. The second assumption brings realism closer to the weak RWR view. The latter, however, does not imply such a hope and, more significantly, does not assume the existence of such properties or the relationships between them, or just a structure, along lines of ontic structural realism.

Thus, classical mechanics (used in dealing with individual objects and small systems, apart from chaotic ones), classical statistical mechanics (used in dealing, statistically, with large classical systems), or chaos theory (used in dealing with classical systems that exhibit a highly nonlinear behavior) are realist theories. While classical statistical mechanics does not represent the behavior of the systems considered because their great mechanical complexity prevents such a representation, it assumes that the individual constituents of these systems are represented by classical mechanics. In chaos theory, which, too, deals with systems consisting of large numbers of atoms, one assumes a mathematical representation of the behavior of these systems. Our phenomenal experience can only serve us partially in relativity. This is because, while we can give the relativistic behavior of photons a concept and represent it mathematically, which makes relativity a realist and causal and, in fact, deterministic theory, we have no means of phenomenally visualizing this behavior or the behavior represented by Einstein’s velocity-addition formula for collinear motion $s=\frac{v+u}{1+{\left(vu/c\right)}^{2}}$. Thus, when the velocity is close to *c* (or is *c*), the relativistic concept of motion is no longer a mathematical refinement of our phenomenal sense and the corresponding ordinary concept of motion in the way the classical concept of motion is. Relativity was the first physical theory that defeated our ability to form a phenomenal conception of individual physical behavior, and as such, it was a radical change in the history of physics. Photons, which only exist in motion with a velocity equal to *c* in a vacuum, represent the limit case. Ultimately, they are quantum entities, which need to be treated by quantum electrodynamics QED. In any event, relativity still offers a conceptual, as well as mathematical, representation of the behavior of individual systems. This behavior could, moreover, be treated causally and indeed deterministically, although, because all physical influences are limited by *c*, relativity imposes new limits on causal relationships between events, by restricting causes to those occurring in the backward (past) light cone of the event that is seen as an effect of this cause, while no event can be a cause of any event outside the forward (future) light cone of that event.

All theories just mentioned are based in the idea that we can observe the phenomena considered without disturbing them sufficiently to affect them [2] (v. 1, p. 53). As a result, one can, for all practical purposes, identify these phenomena with the corresponding objects in nature in their independent behavior and (ideally) represent and predict their behavior by using this representation by these theories, keeping in mind qualifications just made for classical statistical physics or chaos theory. This identification, thus, helps realism, but does not guarantee it, even in the case of classical mechanics, where representational idealizations are more in accord with our phenomenal experience, as I. Kant already realized [13]. In this case, or in relativity, these predictions are still ideally exact, as opposed to the probabilistic or statistical predictions of quantum theory, even in dealing with most elementary individual quantum phenomena. This is a fundamental difference, arising, one is compelled to argue, because of the impossibility of controlling the physical interference of measuring instruments with the object under investigation, in any interpretation of quantum phenomena and QM, or QFT.

The representation of individual physical (quantum) objects and behavior became partial in Bohr’s 1913 atomic theory. The theory only provided representations, in terms of orbits, for the stationary states of electrons in atoms (in which electrons had constant energy levels), but not for the discrete transitions, “quantum jumps,” between stationary states. This was an unprecedented and at the time nearly unimaginable step, because this concept was incompatible with classical mechanics and electrodynamics alike. It was expected that Bohr’s theory was a temporary expedient that would no longer be necessary when a proper theory of quantum phenomena was developed. It was, however, this concept that became central for Heisenberg, who built on it by abandoning an orbital representation of stationary states as well. This led him to his discovery of QM, the first physical theory that allowed for an RWR-type interpretation, at least of the weak type, of it as a whole, as opposed to only partially conforming to the RWR view as Bohr’s 1913 theory was. According to Bohr’s 1925 assessment:

*In contrast to ordinary mechanics, the new quantum mechanics does not deal with a space–time description of the motion of atomic particles. It operates with manifolds of quantities [matrices] which replace the harmonic oscillating components of the motion and symbolize the possibilities of transitions between stationary states …. These quantities satisfy certain relations which take the place of the mechanical equations of motion and the quantization rules.*

[2] (v. 1, p. 48)

Following Heisenberg’s own thinking at the time, this assessment was thus based on the (weak) RWR view and the corresponding interpretation of QM, implicit here rather than developed by Bohr. By contrast, the first worked-out version of Bohr’s interpretation, in his 1927 Como lecture [2] (v. 1, 52–91), restores, ambivalently, realism to QM, by assuming that the independent behavior of quantum objects was represented by the formalism of QM. The Como version of his interpretation was, however, quickly abandoned by Bohr, following his discussion with Einstein in October of 1927 at the Solvay conference in Brussels. This discussion initiated his path toward his ultimate, (strong) RWR-type, interpretation.

This interpretation was first sketched in his 1937 article, “Complementarity and Causality” [14]. Bohr did not use the language of “reality without realism,” but his view, as defined by the irreducible role of measuring instruments in the constitution of quantum phenomena, amounted to the RWR view. He referred to “our not being any longer in a position to speak of the autonomous behavior of a physical object, due to the unavoidable interaction between the object and the measuring instrument,” which, by the same token, entails a “renunciation of the ideal of causality in atomic physics” [14] (p. 87). This and related statements by Bohr could be read as making a stronger claim implying the incapacity of any theory to restore this position to us. I shall, however, view all such statements as belonging to Bohr’s interpretation, from 1937 on, as a strong RWR-type interpretation, placing quantum objects and behavior beyond conception. Bohr clearly does so here. For, if one is no longer in a position to speak of the autonomous behavior of a physical object, this behavior must also be beyond conception, because, if one had such a conception, one would be able to says something about it.

There is still the question of whether our inability to do so only (A): characterizes the situation as things stand now, while allowing that quantum phenomena or whatever may replace them will no longer make this assumption and thus RWR-type interpretations viable, thus reverting to a realist view, or (B): reflects the possibility that this reality will never become available to thought. Logically, once (A) is the case, then (B) is possible too, but is not certain. There does not appear to be any experimental data compelling one to prefer either. (A) and (B) are, however, different in defining how far our mind can, in principle, reach in understanding nature. This is the main reason to distinguish these views, although all of my argument here equally apply to both. Bohr at least assumed (A), and some of his statements, especially those that make stronger than interpretive claims concerning our lack of access to the ultimate nature of reality responsible for quantum phenomena, suggest that he might have entertained (B). The qualification “as things stand now” applies, however, to (B) as well, even though it might appear otherwise given that this view precludes any conception of the ultimate constitution of the reality responsible for quantum phenomena not only now but also ever. It applies because a return to realism in quantum theory is possible, either on experimental or theoretical grounds, if quantum theory, as currently constituted, is replaced by an alternative theory that requires a realist interpretation. This might make the strong (or weak) RWR view obsolete even for those who hold it and is replaced by a more realist view with quantum theory in place in its present form.

One of the reasons for entertaining (B) is that our neurological constitution and, thus, our thinking and language, enabled by this constitution, have evolutionarily developed in our interaction with objects in the world consisting of billions of atoms and thus became essentially classical. Our thinking works in the ways which classical physics mathematically refines, a point often made by Bohr and Heisenberg. Accordingly, there is no special reason to assume that our thought and language should be able to conceive of and describe how nature ultimately works at its very small (or very large) scales.

In either (A) or (B) form, the RWR view, requires a reconsideration of causality and possibly, a “renunciation of the ideal of causality,” as Bohr said, although it is difficult to assume, contrary to Bohr’s claim elsewhere, this renunciation to be “final” [11] (p. 697). As is clear from Bohr’s argument in this article and beyond, this ideal is grounded in the following concept of causality. This concept is defined by the claim that the state, X, of a physical system is definitively (rather than with any probability other than equal to one) determined, in accordance with a law, at all future moments of time once it is determined at a given moment of time, state A, and A is determined definitively in accordance with the same law by any of the system’s previous states. This assumption, thus, implies a concept of reality, which defines this law, thus making this concept of causality ontological. By making such a concept inapplicable to the ultimate constitution of the reality responsible for quantum phenomena, the RWR view precludes the application of causality to the relationships between quantum phenomena or events.

Certain qualifications of this definition of causality are in order, however. In particular, this definition, which does not use the concept of cause but only that of the exact determination according to a given law, need not imply that A is a cause of X, in accord, say, with Kant’s understanding of causality. This understanding has been commonly used in considering causality since Kant, a key figure in the modern history of the question of causality, although what he defines as the principle of causality has been commonly used earlier, beginning with Plato and Aristotle, or even the pre-Socratics. Kant defined the principle of causality as follows: if an event takes place, it has, at least in principle, a cause of which this event is an effect defined, inevitably and strictly, by a given rule or law [13] (pp. 305, 308). It is commonly (although there are exceptions) assumed that the cause must be prior to, or at least simultaneous with, the effect, an assumption also known as the antecedence postulate. Now, the fact that the physical state of a body at point *t*_1_ determines, by Newton’s law of gravity, the state of this body at any other point *t*_2_ does not mean that the state at *t*_1_ is the cause of the state at *t*_2_. One might argue that the real physical cause for any determination, including that of the initial state, A, that defines any particular case considered, is (in our language) the gravitational field defined by the Sun and other physical objects in the Solar system, as encoded in Newton’s law of gravity. In this view, a given state, A, of any single object can only be seen as a physical cause of its future states, insofar as the whole configuration of bodies and forces involved, which determines the law of motion and hence causality, is viewed as embodied in this state. The history of the system thus considered, or any of classical system, whatever a physical law or set of laws defines its causal behavior, only goes so far in a given representation, which suspends more remote causes, let alone of the ultimate cause of this history. (Assuming the ultimate cause of anything is a major philosophical problem, put aside here.) Thus, Newton bracketed the physical nature of and the causes of gravity and was (wisely) content to merely define a law of gravity in considering, as causal, the behavior of any given object under this law, and other laws of Newton’s mechanics. In considering planetary motion, all history, such as that of the emergence of the Solar system, was bracketed as well. This type of bracketing is workable on very large spatial and temporal scales, even that of the Universe itself, using Newton’s theory of gravity or general relativity, but only *up to a point*. The situation changes once one gets closer to the Big Bang or to considering the Big Bang itself (assumed to be a complex process in which great many things happen even if in very short time, by our measure), because of the quantum aspects and possible still other have to be considered. This leads to complexities that thus far has defeated all our efforts of resolving them.

For the moment, a well-defined situation in physics (classical, relativistic, or quantum) usually entails the cut off defined by the initial state and the actual or possible final state of the system considered, with either one or both determined by measurements we do or can perform. When at stake are predictions, this demarcation is defined by the initial measurement, performed by us, possibly using nature as part of our observational technology and a possible future measurement that can verify the prediction made. Such predictions can only be made by us, even when they concern the behavior of objects in accordance with causality and, in the case of some causal systems, are exact or deterministic. Nature does not make predictions. In the present view, it does not make measurements and has no quantum objects, like elementary particles, either. Perhaps, as discussed in Section 4, it has quantum fields, that is, such a concept may reflect better the ultimate constitution of nature or part of this constitution responsible for quantum phenomena.

The view of classical physics or relativity, or even quantum physics as conforming by the concept of causality as defined above (which need not require the idea of cause, but only the law strictly connecting events and enabling exact predictions), has been nearly universally accepted. Quantum theory, and especially Bohr’s 1913 atomic theory, introduced in the same year, radically challenged this view. As Bohr said later, “The unrestricted applicability of the causal mode of description to physical phenomena has hardly been seriously questioned until Planck’s discovery of the quantum of action” [15] (p. 94). Quantum phenomena, at least in the RWR view, expressly violate the principle of causality because no determinable event (or phenomenon) could be established as the cause of a given event (or phenomenon), and only statistical correlations between events could be ascertained. But then, there is, in the RWR view, no relation of the type corresponding to causality as defined here, although claims concerning the existence of such relations are found in considering QM and QFT. In the RRW view, the equations of QM or QFT, such as Schrödinger’s or Dirac’s equation, only provide, with the help of Born’s rule, the probabilities of the outcomes of possible future quantum experiments of the basis of previously performed ones. Born’s rule or an analogous rule (such as von Neumann’s projection postulate or Lüder’s postulate), establishes the relation between the so-called “quantum amplitudes,” associated with complex Hilbert-space vectors as complex entities and probabilities as real numbers, by using square moduli or, equivalently, the multiplication of these quantities and their complex conjugates (technically, these amplitudes are first linked to probability densities). Although Born’s or analogous rules are connected naturally to the formalism of QM, they are added to this formalism rather than are derived from it. We do not know why these postulates work, which makes it tempting to argue that why they work is the greatest mystery of QM, but then, we do not know why the formalism works either. There is neither one without the other.

Some, beginning with P. S. Laplace, have used “determinism” for causality in the present sense. His and related views may be seen as forms of ontological determinism. Laplace (one of the founders of modern probability theory) and others who have adopted such views were of course aware of the necessity of using of probability in physics but only assume it to be necessary for practical, epistemological reasons, due to our lack of knowledge concerning causal or deterministic relationships ultimately defining all events considered. In part for this reason, I prefer to define “determinism” as an epistemological category referring to the possibility of predicting the outcomes of causal processes ideally exactly in accordance with laws that define them as causal. In classical mechanics, when dealing with individual objects or small systems (apart from chaotic ones), both concepts in effect coincide or rather, because they are different concepts, are correlatively applicable. On the other hand, classical statistical mechanics or chaos theory are causal but not deterministic in view of the complexity of the systems considered, which limit us to probabilistic or statistical predictions concerning their behavior.

In the case of quantum phenomena, deterministic predictions are not possible on experimental grounds even in considering the most elementary quantum phenomena, such as those associated with elementary particles. This is because the repetition of identically prepared quantum experiments in general leads to different outcomes, and unlike in classical physics, this difference cannot be diminished beyond the limit defined by Planck’s constant, *h*, by improving the capacity of our measuring instruments. This impossibility is manifested in the uncertainty relations, which would remain valid even if we had perfect instruments and which pertain to the data observed, rather than to any particular theory. Hence, the probabilistic or statistical character of quantum predictions must also be maintained by interpretations of QM or alternative theories of quantum phenomena that are causal. Such interpretations and theories are also, and in the first place, realist because causality implies a law governing it and thus a representation of the reality considered (in these cases, defined by the behavior of quantum objects) in terms of this law. By contrast, RWR-type interpretations are not causal because of the absence of realism in considering the ultimate nature of reality responsible for quantum phenomena.

The meanings of the terms causality and determinism fluctuate in physical and philosophical literature. Thus, Schrödinger’s or Dirac’s equation is sometimes seen as “deterministic” or “causal” under the assumption that it describes, even in a causal way, the independent behavior of quantum objects, with the recourse to probability only arising because of the interference of measurements into this behavior. This assumption is shared by both von-Neumann’s and Dirac’s classic books [16,17,18] and ambivalently by Bohr in his Como lecture [18] (pp. 191–218). It poses difficulties, beginning with the fact that the variables involved are complex quantities in Hilbert spaces over ℂ. These difficulties were confronted by E. Schrödinger because his wave-equation was dealing with waves in the configuration space rather than physical space. I shall put these difficulties aside here because they do not affect my argument. In RWR-type interpretations, either equation only determines the *mathematical* state, the “quantum state,” of the corresponding wave-function as a Hilbert-space vector at any future point once it is determined at a given point, *mathematically*. It would be more accurate to say that each equation determines it for any given value of the parameter *t* in the equation, which values could be related to time-measurements at different points in time. The relationships between these measurements themselves are probabilistic, which also suggests a possibility of conceiving of quantum states themselves in probabilistic terms (e.g., [19]) or redefining causality itself as a probabilistic concept (e.g., [7], pp. 1844–1846; [20]), a subject that, however, requires a separate consideration. Accordingly, *physically*, each equation only determines, in Schrödinger’s terms, an “expectation-catalog” concerning the outcomes of possible future experiments, to be observed in measuring instruments, without representing either how they come about or these outcomes themselves, represented by classical physics [21] (p. 154). Hence, contrary to another common claim, neither equation is seen as time-reversible either.

I comment next on indeterminacy, randomness, and probability from the RWR perspective. I reiterate first that the strong RWR view makes the absence of causality automatic because assuming the ultimate character of the reality responsible for quantum phenomena to be causal would imply at least a partial conception or even representation of this reality as concerns the law that governs it and causally connects events. This does not mean that interpretations of QM or alternative theories of quantum phenomena that are realist and causal are impossible. Even in this case, however, and assuming that quantum objects exist independently (rather than, as in the present view, only at the time of measurement), one cannot track them individually in the way one can individual classical objects by separating their behavior from their interaction with measuring instruments, which implies that the difference between objects and phenomena remains irreducible in this case as well. It follows that, although causality, or in the first place, realism, is possible in considering quantum phenomena, determinism is not. Accordingly, while in classical physics or relativity, where all systems considered are causal, some of them are handled deterministically and others probabilistically or statistically, all quantum systems can only be handled probabilistically or statistically, even if one assumes causality.

I shall now define the concepts of indeterminacy, randomness, chance, and probability, as they are understood here, because their meanings fluctuate as well. In the present definition, indeterminacy is a more general category, while randomness or chance will refer to a most radical form of indeterminacy, when a probability cannot be assigned to a possible event, which may also occur unexpectedly. Randomness and chance may also be understood as different from each other. These differences are, however, not germane in the present context, and I shall only speak of randomness. Both indeterminacy and randomness only refer to possible future events and define our expectations concerning them. Once an event has occurred, it is determined. An indeterminate nature of events may either allow for assuming an underlying causal architecture of the physical reality responsible for this nature, whether this process is accessible to us or not, or disallow for making such an assumption. The first case defines indeterminacy in classical physics, in particular classical statistical physics or chaos theory, and the second in QM, in RWR interpretations. It is impossible to ascertain that an apparently random sequence of events, events that occurred apparently randomly, was in fact random, rather than connected by some rule, such as that defined by causality, and there is no mathematical proof that any sequence is. The sequences of indeterminate events that allow for probabilistic predictions concerning them is a different matter, although there is still no guarantee that such sequences are not ultimately underlain by causal connections in the case of quantum phenomena. Experimentally, as explained, quantum phenomena only preclude determinism, because identically prepared quantum experiments, as concerns the state of measuring instruments, in general lead to different outcomes. Only the statistics of multiple (identically prepared) experiments are repeatable, which is fortunate, because otherwise it would be impossible to have a scientific theory, such as QM or QFT, of these data.

A Bayesian view of quantum theory, based on dealing with probabilities of individual events, would qualify the situation, by making QM or QFT probabilistic, rather than statistical, while, however, retaining the fundamental difference in question between quantum and classical physics and the possibility of using the statistical data of either theory. First, “probabilistic” commonly refers to our estimates of the probabilities of either individual or collective events, such as that of a coin toss or of finding a quantum object in a given region of space. “Statistical” refers to our estimates concerning the outcomes of identical or similar experiments, such as that of multiple coin-tosses or repeated identically prepared experiments with quantum objects, or to the average behavior of certain objects or systems. There are different versions of the Bayesian view (e.g., [22,23]). Most generally, however, it defines probability as a degree of belief concerning a possible occurrence of an individual event on the basis of the relevant information we possess. This makes probabilistic estimates, generally, subjective, although there may be agreement (possibly among a large number of individuals) concerning such estimates. The frequentist understanding, also revealingly referred to as “frequentist *statistics*,” defines probability in terms of sample data by emphasis on the frequency or proportion of these data, which is considered more objective. In quantum physics, exact predictions are, again, impossible even in dealing with elemental individual processes and events. This fact could, however, be interpreted either on Bayesian lines, under the assumption that a probability could be assigned to individual quantum events, or on frequentist lines, but under the assumption that each individual effect is strictly random and hence cannot be assigned a probability at all. (The standard use of the term “quantum statistics” refers to the behavior of large multiplicities of identical quantum objects, such as electrons and photons, which behave differently, in accordance with the Fermi–Dirac and the Bose–Einstein statistics, for identical particles with, respectively, half-integer and integer spin).

An example of a Bayesian approach, which is of an RWR-type in the present definition, is Quantum Baeysianism, or QBism [24,25]. I qualify because, QBists themselves sometimes speak of realism by virtue of assuming the existence of exterior physical reality [24]. Although my argument in this article would equally apply if one assumes a Bayesian, RWR-type, view, I adopt the frequentist, RWR-type, view, considered in detail in [4] (pp. 173–186), [8]. Bohr appears to have been inclined to a statistical view as well [2] (v. 2, pp. 18), [4] (pp. 180–184). I might add that, while I can understand why QBism sees our assignments of probabilities as subjective, I would prefer to say that these assignments human, which makes all probabilistic relationships between a given theory and observed phenomena human as well, in classical (or relativistic) and quantum physics alike. Nature does not assign probabilities. In classical physics (or relativity), however, these relationships may be, ideally, assumed to be *physically* grounded in the behavior of the system considered, because classical physics may be assumed to represents this behavior, which is no longer possible to assume in quantum theory, at least in RWR-type interpretations. There have been statistical interpretations of QM, commonly on realist lines. Two instructive examples are those of A. Khrennikov [8,26] and A. E. Allahverdyan, R. Balian, and T. Nieuwenhuizen [27]. While Khrennikov’s interpretation is expressly realist, that of Allahverdyan, Balian, Nieuwenhuizen may be seen as allowing for RWR-type interpretations. This is because they argue that one should only interpret outcomes of pointer indications and leave the richer quantum structure, which has many ways of expressing the same identities, without interpretation. In RWR-type interpretations, this structure would be seen as enabling statistical predictions, without representing the ultimate constitution of the reality responsible for the outcomes of experiments and thus pointer indications.

Probability introduces an element of order into situations defined by the role of indeterminacy in them and enables us to handle such situations better. Probability or statistics is about the interplay of indeterminacy and order. This interplay takes on a unique significance in quantum physics, because of the existence of quantum correlations, such as the EPR (Einstein–Podolsky–Rosen) or, in the case of discrete variables, EPR-Bell correlations. These correlations are properly predicted by QM, which is, thus, as much about order as about indeterminacy, and about their unique combination in quantum physics. The correlations themselves are collective, statistical, and thus do not depend on either the Bayesian or frequentist view of the individual events involved.

The circumstances just outlined imply a different reason for the recourse to probability in quantum physics, in RWR-type interpretations. According to Bohr, the idea of indeterminacy apart from “the causal mode” of understanding reality has “hardly been seriously questioned until Planck’s discovery of the quantum of action” [17] (p. 94). As he said on a later occasion (in 1949): “[E]ven in the great epoch of critical [i.e., post-Kantian] philosophy in the former century, there was only a question to what extent a priori arguments could be given for the adequacy of space-time coordination and causal connection of experience, but never a question of rational generalizations or inherent limitations of such categories of human thinking” [2] (v. 2, p. 65). Even more radical philosophical questionings of causality, such as those by D. Hume, are those of our epistemological capacity to grasp the underlying causal order presupposed at the ultimate level of reality. According to Bohr:

*[I]t is most important to realize that the recourse to probability laws under such circumstances is essentially different in aim from the familiar application of statistical considerations as practical means of accounting for the properties of mechanical systems of great structural complexity [in classical physics]. In fact, in quantum physics we are presented not with intricacies of this kind, but with the inability of the classical frame of concepts to comprise the peculiar feature of indivisibility, or “individuality,” characterizing the elementary processes.*

[2] (v. 2, p. 34)

Rather than representing a definitive state of affairs in nature, even if Bohr thought so, this statement should be seen as expressing the strong RWR-type *interpretation* adopted by Bohr at this point, in 1949. For one thing, as noted, some interpretations of QM, such as those by von Neumann [16] and Dirac [17], or alternative theories, such as Bohmian mechanics, assume causal views of the behavior of quantum objects, with probability or statistics brought in by measurement. Individuality and indivisibility reflect the features of Bohr’s concept of phenomena. Referring to “the elementary processes” is due to the fact, in place from Planck’s discovery of quantum theory on, that exact predictions are no longer possible, even ideally, regardless of how elementary quantum objects may be.

“The classical frame of concepts” may appear to refer to the concepts of classical physics, and it does include these concepts. By this time (in 1949), however, Bohr adopts the strong RWR view, which places the ultimate nature of reality responsible for quantum phenomena and possible all physical phenomena beyond conception. This gives the phrase “the classical frame of concepts” a broader meaning: all representational concepts that we can form are classical or proto-classical. They may be seen as proto-classical insofar as the physical concepts of classical physics and, as noted, already with significant limitations, relativity may be considered as refinements of our phenomenal intuition, a product of our evolutionary neurological machinery, intuitions embodied in ideas like bodies and motion. This refinement is no longer available for representing the ultimate nature of reality responsible for quantum phenomena or possibly the ultimate constitution of nature, at least as things stand now. Classical physical concepts are, however, still used in quantum physics in RWR-type interpretations, in particular that of Bohr or the present one, in dealing with the behavior of the observable parts of measuring instruments and, thus, data or information found in these parts. But these concepts, again, do not, in these interpretations, apply to the ultimate character of physical reality responsible for quantum phenomena. That still need not mean that a realist interpretation of quantum phenomena or QM or an alternative theory of quantum phenomena, using “the classical frame of concepts,” is impossible. As stated above, the RWR view may become obsolete in quantum theory in its present form or whatever may replace it even for those who hold it and replaced by a more realist view, based “on the classical frame of concepts.” In other words, even if the ultimate constitution of nature is still assumed to be beyond representation or even conception, because our own evolutionary neurological constitution limits us to classical or proto-classical frame of concepts, there will be no physics that needs to take this limitation into account as concerns any physical phenomena.

For the moment, in RWR-type interpretations, only two types of concepts are available for dealing with the ultimate nature of reality responsible for quantum phenomena, while the classical frame of concepts is used for describing the observable parts of measuring instruments and quantum phenomena, or data or information registered in them. The first type are purely mathematical concepts. Their role eventually led Heisenberg to a form of mathematical realism, with a Platonist flavor, while assuming that QM or QFT does not represent quantum objects and behavior by physical concepts, at least as we conventionally understand them, for example, in classical physics or relativity [28] (pp. 145, 167–186). By contrast, in his ultimate (strong RWR-type) interpretation, Bohr rejected the possibility of a mathematical representation, along with a physical one, of the ultimate nature of reality responsible for quantum phenomena, at least as things stand now. The present interpretation is in accord with Bohr on this point.

The second type of concepts are physical concepts, defined here as RWR-concepts, such as Bohr’s concept of phenomena and complementarity, when complementarity is that of phenomena. These concepts have both representational (possibly classical) components and RWR-components and thus reflect that the ultimate nature of reality responsible for quantum phenomena is beyond representation or conception, which defines their RWR-components. This structure is in accord with and is physically defined by the two-component structure of measuring instruments, consisting in their classical describable observable part and their quantum strata through which they interact with the ultimate, RWR-type, physical reality responsible for quantum phenomena. According to Bohr:

*I advocated the application of the word *phenomenon* exclusively to refer to the observations obtained under specified circumstances, including an account of the whole experimental arrangement. In such terminology, the observational problem is free of any special intricacy since, in actual experiments, all observations are expressed by unambiguous statements referring, for instance, to the registration of the point at which an electron arrives at a photographic plate. Moreover, speaking in such a way is just suited to emphasize that the appropriate physical interpretation of the symbolic quantum-mechanical formalism amounts only to predictions, of determinate or statistical character, pertaining to individual phenomena appearing under conditions defined by classical physical concepts [describing the observable parts of measuring instruments].*

[2] (v. 2, p. 64)

Referring to “observations” is precise, because only the classically observed properties of measuring instruments affected by these observations could be measured. (By a “quantum measurement” I, again, refer to this whole process.) As defined by “*the observations [already] obtained under specified circumstances*,” phenomena refer to events that have already occurred, and not to future events that one can predict on the basis of previous events defined by already established phenomena. This is a crucial point, discussed further in Section 3. Referring, phenomenologically, to observations also explains Bohr’s choice of the term “phenomenon.” This idealization is the same as that of classical physics, which allows one to identify phenomena with the physical objects (here measuring instruments), because an observations does not interfere with their behavior, in contrast to the way an observation by means of a measuring instrument interferes with the ultimate constitution of the reality responsible for a phenomenon thus observed in quantum physics. On the other hand, given that a quantum object is, in the present view, an idealization applicable only at the time of measurement, it is a product of this interference.

Complementarity adds a new dimension to this situation. Complementarity is defined by

(a) A mutual exclusivity of certain phenomena, entities, or conceptions, such as, and in particular, those of the position and the momentum measurements, which can never be performed simultaneously in view of the uncertainty relations;
(b) The possibility of considering each one of them separately at any given point;
(c) The necessity of considering all of them at different moments of time for a comprehensive account of the totality of phenomena that one must consider in quantum physics.

In Bohr’s ultimate, strong RWR-type, version of his interpretation, complementarity applies to quantum phenomena observed in measuring instruments. Each of the two complementary phenomena involved in a given complementarity, say, that of the exact position or the exact momentum measurement, associated with a quantum object, may be established alternatively at any given point in time. They cannot, however, be both established (exactly) simultaneously. Neither concept, phenomenon or complementarity, represents the ultimate nature of reality responsible for quantum phenomena. They reflect the impossibility of representing it.

## 3. Measurement, Idealization, and Quantum Indefinitiveness

This section explains in detail and derives implications from the tripartite structure of the idealization of physical reality assumed by the present view of quantum measurement. As stated from the outset, this structure is not inherent in and is not necessarily adopted by RWR-type interpretations of quantum phenomena and QM or QFT, including that of Bohr. Even if, given some of his statements, it might be seen as a consequence of Bohr’s view, Bohr never expressly stated this consequence. Bohr says, for example, that “the concept of stationary states may indeed be said to possess, within its field on application, just as much, or, if one prefers, as little ‘reality’ as the elementary particles themselves. In each case, we concerned with expedients which enable us to express in the consistent manner essential aspects of the phenomena” [2] (v. 1, p. 12). That may be close to the present view, and it is clearly a form of the RWR view. The statement still appears, however, more likely to imply that either concept is an idealization but not that the concept of the elementary particle or quantum object, as an idealization, applied only at the time of measurement. According to Fine, commenting on Bohr’s reply to EPR [11]: “But should we say that an electron is nowhere at all until we are set up to measure its position, or would it be inappropriate (meaningless?) even to ask?” [29]. In the present view, an electron is not assumed to have existed, or the corresponding idealization to apply, before the interaction between the ultimate constitution of the reality responsible for quantum phenomena and the measuring instrument. Nevertheless, this argument still builds on Bohr’s argument concerning the irreducible role of measuring instruments in the constitution of quantum phenomena and thus the irreducible difference between them and quantum objects, and subtler aspects of this situation, as suggested by the following elaboration. Bohr says:

*This necessity of discriminating in each experimental arrangement between those parts of the physical system considered which are to be treated as measuring instruments and those which constitute the objects under investigation may indeed be said to form a principal distinction between classical and quantum-mechanical description of physical phenomena. It is true that the place within each measuring procedure where this discrimination is made is in both cases largely a matter of convenience. While, however, in classical physics the distinction between object and measuring agencies does not entail any difference in the character of the description of the phenomena concerned, its fundamental importance in quantum theory … has its root in the indispensable use of classical concepts in the interpretation of all proper measurements, even though the classical theories do not suffice in accounting for the new types of regularities with which we are concerned in atomic physics. In accordance with this situation there can be no question of any unambiguous interpretation of the symbols of quantum mechanics other than that embodied in the well-known rules which allow us to predict the results to be obtained by a given experimental arrangement described in a totally classical way.*

[11] (p. 701)

Before I discuss this elaboration as such, I would like to address two common misunderstandings to which this and related statements by Bohr have often led. First, Bohr’s statement may suggest that, while observable parts of measuring instruments are described by means of classical physics, the independent behavior of quantum objects is described or represented by means of the quantum-mechanical formalism. This type of view has been adopted by some, for example, as noted earlier, von Neumann [16] and Dirac [17], and, in part under the impact these books, it is sometimes referred to as “the Copenhagen interpretation.” It was not, however, Bohr’s view, at least after he revised his Como argument, which entertained this type of view and which had influenced others, including Dirac and von Neumann, in this regard. Bohr *does say* here that the observable parts of measuring instruments are described by means of classical physics and that classical theories cannot suffice to account for quantum phenomena, but he *does not say* that the independent behavior of quantum objects is described by the quantum-mechanical formalism. His statement only implies that quantum objects cannot be treated classically, for if they could be, classical theories would suffice in accounting for the new types of regularities in question. The “symbols” of quantum-mechanical formalism are assumed here, as they always are by Bohr, only to have a probabilistically or statistically predictive role.

Bohr’s insistence on the indispensability of classical physical concepts in considering the measuring instruments is often misunderstood as well, in particular by disregarding that measuring instruments contain both classical and quantum strata. Even though what is observed as phenomena in quantum experiments is beyond the capacity of classical physics to account for them, the classical description can and, in order for us to be able to give an account of what happens in quantum experiments, must apply to the observable parts of measuring instruments. The instruments, however, also have a quantum stratum, through which they interact with quantum objects or, in the present view, the ultimate constitution of the reality responsible for quantum phenomena, which interaction would not be possible without this quantum stratum. The interaction is quantum and thus cannot be observed or, in RWR-type interpretations, represented. It is “irreversibly amplified” to the classical level of observable effects, say, a spot left on a silver screen [2] (v. 2, p. 73). The nature of this “amplification” is a separate matter and is part of the problem, commonly, including by this author, seen as unsolved (although there are claims to the contrary, for example, along the lines of the consistent histories approach), of the transition from the quantum to the classical, which and related subjects, such as “decoherence,” are beyond my scope.

It might be added that one could attempt to formalize this situation, as, for example, in [30,31]. One considers a compound quantum system, QO + QI, consisting of the quantum object under investigation, QO and the quantum part, QI, of the instrument I, QO + QI, which is isolated during the (short) time interval when the quantum interaction in question takes place. The rest of the instrument, I, performs the measurement, a pointer measurement, on QI, after the interaction has taken place. In realist schemes, such as that of M. Ozawa [31], the evolution of the QO + QI, the unitary evolution operator,

$$U\left(t\right)=e^{-i\Delta tH},$$

where

H = H_QO_ + H_QI_ + H_QOQI_

is the Hamiltonian representing the internal behavior of the subsystems involved and H_QOQI_ the interaction between them. In RWR view, no element of the formalism represents the ultimate nature of reality responsible for quantum phenomena, including its stratum involved in the interaction between QO and QI, responsible for the effects observed. Any such element only serves as part of the mathematics of QM that, with the help of Born’s rule, predicts such effects.

The situation under discussion is sometimes referred to as the arbitrariness of the “cut” or, because the term cut [*Schnitt*] was favored by Heisenberg and von Neumann, the “Heisenberg–von-Neumann cut.” As Bohr noted, however, while “it is true that the place within each measuring procedure where this discrimination [between the object and the measuring instrument] is made is … largely a matter of convenience,” it is true only largely, but not completely. This is because “in each experimental arrangement and measuring procedure we have only a free choice of this place within a region where the quantum-mechanical description of the process concerned is effectively equivalent with the classical description” [11] (p. 701). In other words, the ultimate constitution of the physical reality responsible for quantum phenomena, including quantum objects observed in measuring instruments is always on the other side, never the measurement side, of the cut. Neither are quantum strata of the instruments through which the latter interact with this reality. By contrast, the measurement side of the cut is constituted by the effects observed in measuring instruments as the outcomes of quantum experiments, as quantum phenomena, effects that can be represented, moreover, by means of classical physical concepts.

In the present view, while a measuring instrument, which is, in its observable part, a classical object, or, at the other pole, the ultimate constitution of the reality considered, are assumed to be independent, a quantum object can only be rigorously ascribed existence and be defined by a measurement and its setup, including the cut. Accordingly, in this view, there is no independent behavior of quantum objects either: there is only the interaction between the ultimate (RWR-type) nature of reality and measuring instruments, which interaction allows one to define quantum objects. As discussed in next section, this interaction actually takes place before the measurement itself, which pertain to the state of the quantum stratum of the instrument after this interaction, and no longer the quantum object, which thus no longer exist either in the present view [2] (v. 2, p. 57). It is this state, rather than the state of quantum object or the independent reality which the instrument had interacted but no longer does that is then “irreversibly amplified” to the macroscopic, classical level of observable effects, such as a spot left on a silver screen. If one assumes the independent existence of quantum objects, then one can say that “[the quantum object] is already on its way from one instrument to another” [2] (v. 2, p. 57). What is a quantum object in a given experiment can be different in each case, including possibly something that, if considered by itself, could be viewed as classical, as in the case of Carbon 60 fullerene molecules, which were observed as both classical and quantum objects [32]. The quantum nature of any quantum object is still defined by its microscopic constitution.

The following question might, then, be asked. If a quantum object is only (an idealization) defined by an experiment or measurement, rather than as something that exists independently, could one still speak of the same quantum object, say, the same electron, in two successive measurements? For, if the idealization of quantum objects is only applicable at the time of measurement, then a prediction based on a given measurement and the new measurement based on this prediction could only concern a new quantum object, arising in the interaction between the ultimate constitution of the reality responsible for quantum phenomena and measuring instruments and not an object measured earlier in making a prediction. Accordingly, in the present view, rigorously, one deals with two different quantum objects, two different electrons, for example. To consider them as the same electron is, however, a permissible idealization in low-energy (QM) regime, an idealization ultimately statistical in nature, because a collision with the screen, after the electron passes the slit, is not guaranteed, although the probability that it will not occur is low. Nevertheless, one could still, within these limits, speak of the transition between two (physical) states of the same quantum object, with each state defined by the effects observed in measuring instruments. On the other hand, as discussed in Section 5, speaking of the same electron in any two successive measurements in high-energy (QFT) regimes is meaningless, which further justifies the present concept of a quantum object and the tripartite idealization scheme, adopted here.

The epistemological cost of the RWR view is not easily absorbed by most physicists and philosophers, and to some, beginning, famously, with Einstein, is unacceptable. Both Schrödinger and Bell are among prominent figures and symbols of this resistance, as is of course, even more so, Einstein. This attitude is not surprising because the features of quantum phenomena that are manifested in many famous experiments and that led to RWR-views defy many assumptions concerning nature commonly considered as basic. These assumptions, arising due to the neurological constitution of our brain, have served us for as long as human life, and within certain limits, are unavoidable, although, while fully respected by classical physics, their scope, was already challenged by relativity. QM have made this challenge much greater. As noted earlier, however, the same neurological constitution may also prevent us from conceiving of the ultimate (RWR-type) nature of physical reality responsible for quantum phenomena. Thus, it is humanly natural to assume that something happens between observations. The sense that something happened is one of the most essential elements of human thought. However, in the RWR view, the expression “something happened” is ultimately inapplicable to the ultimate constitution of the reality responsible for quantum phenomena. According to Heisenberg:

*There is no description of what happens to the system between the initial observation and the next measurement. …The demand to “describe what happens” in the quantum-theoretical process between two successive observations is a contradiction in adjecto, since the word “describe” refers to the use of classical concepts, while these concepts cannot be applied in the space between the observations; they can only be applied at the points of observation.*

[28] (pp. 57, 145)

The same would apply to the word “happen” or “system,” or any word we use, whatever concept it may designate, including reality, although when “reality” refers to that of the RWR-type, it is a word without a concept attached to it. As Heisenberg says: “But the problems of language are really serious. We wish to speak in some way about the structure of the atoms and not only about ‘facts’—the latter being, for instance, the black spots on a photographic plate or the water droplets in a cloud chamber. However, we cannot speak about the atoms in ordinary language” [28] (pp. 178–179). Nor is it possible in terms of ordinary concepts, from which ordinary language is indissociable, or, in the RWR view, even in terms of physical concepts, assuming the latter can be entirely dissociated from ordinary concepts.

This is a formidable problem even if one adopts the strong RWR view. The term “reality” in the phrase “reality without realism” does not pose a difficulty here, because this term has no concept associated to it, making it akin to a mathematical symbol. A greater difficulty are expressions like “quantum objects interact with each other,” used for example, in considering the EPR experiment and entanglement, or “the interaction between the independent RWR-type reality and a measuring instrument,” which refer to something between or before observations. One can handle this difficulty as follows in the RWR view. Although one can provisionally speak of a “relation” between two or more quantum objects, there is no term or concept, such as “interaction” or “relation,” or “taking place,” applicable to what “takes place.” Any rigorous statement can only concern observable events, with which, moreover, and only with which the concept of a quantum objects is associated. Accordingly, one cannot rigorously speak of an interaction between quantum objects between experiments, with the concept a quantum object itself only applicable at the time of measurement in the present interpretation. One can only speak of two quantum objects associated with two measurements performed initially and then two quantum objects associated with two measurements performed subsequently. These measurements may be related in one way or another, for example, in terms of entanglement, and predicted accordingly, in the case of entanglement, by using the concept of an entangled states, in the formalism. Mathematical concepts are, in Heisenberg’s view, a possible exception, which I shall consider presently.

Before I do so, I would like to formulate the quantum indefinitiveness postulate, which is a consequence the RWR view and reflects the situation just considered. It dictates *the impossibility of making definitive statements of any kind, including mathematical ones, concerning the relationship between any two individual quantum phenomena or events, indeed to definitively ascertain the existence of any such relationship*. It does allow for making definitive statements concerning individual phenomena or events, defined by measurements, and statements (statistical in nature) concerning the relationships between multiple events. It only concerns events that have already happened, rather than possible future events, in which case one can make probabilistic statements, on Bayesian lines.

Precluding the possibility of any mathematical connections between individual events makes the postulate stronger than Heisenberg’s claim, cited above. While prohibiting a common-language and in effect physical description of what “happens” between quantum experiments, this claim in principle allows for the *mathematical representation* of what the ultimate constitution of physical reality, including, in Heisenberg’s view in his later works. The word “happens” or even “physical” need, accordingly, no longer applies to this representation. As Heisenberg said on an earlier occasion, mathematics is “fortunately” free from the limitations of ordinary language and concepts:

*It is very difficult to modify our language so that it will be able to describe these atomic processes, for words can only describe things of which we can form mental pictures, and this ability, too, is a result of daily experience. Fortunately, mathematics is not subject to this limitation, and it has been possible to invent a mathematical scheme—the quantum theory [e.g., QM]—which seems entirely adequate for the treatment of atomic processes.*

[33] (p. 11).

In physics, however, mathematics enables us to *relate* to things in nature which are beyond the reach of our thinking, including mathematical ones. In quantum theory, it does so by enabling us to estimate probabilities or statistics of quantum events, to which Heisenberg refers here by speaking of this scheme, QM, as “entirely adequate for the treatment of atomic processes.” At the time, Heisenberg, adopting the RWR view, used this freedom to construct QM as a theory only designed to predict the probabilities or statistics of events observed in measuring instruments. It is in fact equally fortunate that nature allows us, in our interaction with it, just to have such a scheme, for the fact of its freedom from the limitations of common language and concepts, or in its abstract nature, even physical concepts, does not guarantee that it will work in physics. By contrast, in his later writings, in part in view of QFT, Heisenberg assumed the possibility of a mathematical *representation* of the ultimate constitution of reality, while excluding physical concepts (at least in their customary sense found in classical physics or relativity) as applicable to this constitution [28] (pp. 145, 167–186). Heisenberg speaks of this representation in terms of symmetry groups and defines elementary particles accordingly, without considering them as particles in a physical sense. The concept of elementary particle can be given a mathematical sense insofar as the corresponding representation of the group is irreducible [34,35]. Heisenberg even suggests that Kant’s thing-in-itself is “finally, a mathematical structure:” “The ‘thing-in-itself’ is for the atomic physicist, if he uses this concept at all, finally a mathematical structure; but this structure is—contrary to Kant—indirectly deduced from experience [rather than is given to our thought a priori, as in Kant]” [28] (p. 83).

Bohr, by contrast, rejected the possibility of a mathematical representation of quantum objects and behavior, or the reality they idealize, along with a physical one, at least in his ultimate, strong RWR-type, interpretation. It is true that Bohr often speaks of this reality as being beyond our phenomenal intuition, also involving visualization, sometimes used, including by Bohr, to translate the German word for intuition, *Anschaulichkeit* (e.g., [2] (v. 1 p. 51, 98–100, 108; v. 2, p. 59)). It is clear, however, that, apart from the Como lecture, Bohr saw the ultimate nature of this reality as being beyond any representation or even conception, including a mathematical one, at least as things stand now.

Indeed, notwithstanding its dominant role in modern physics, amplified and even made unique by in quantum theory, it is not clear why mathematics, which is the product of the same human thinking as ordinary language or physical concepts are, should be able to represent how nature ultimately works at its very small (or very large) scales. It is not clear either that, in contrast its capacity to do so at the scales handled by QFT, mathematics will enable us to predict phenomena shaped by the workings of nature far at the smaller scales, such as Planck’s scale, although the current consensus is that it should be able to do so. However, a consensus is not always a guarantee. Bohr, in speaking of “the special role … played by mathematics in development of [all] logical thinking” and “invaluable help [offered] by its well-defined abstractions” in quantum theory, nevertheless, added: “Still, … we should not consider pure mathematics as a separate branch of knowledge, but rather as a refinement of general language, supplementing it with appropriate tools to represent relations for which ordinary verbal expression is imprecise and cumbersome” [2] (v. 2, p. 68). This refinement can take us very far from ordinary language and concepts, the distance that is, again, manifested in the mathematics of QM and QFT, as Bohr was well aware. Nevertheless, mathematics is still human. As such, it may not ultimately be suited, because nothing human can, to deal with the ultimate constitution of nature, either in terms of representing or conceiving of it or even only in predicting probabilistically, as QM or QFT does, the outcome of the events considered. I am not saying that we cannot go further with our fundamental physical theories and their mathematics; quite the contrary, especially given the history of quantum theory, which is the affirmation of mathematical thinking in physics. This thinking is all the more remarkable because it connects us, by means of mathematics, to that which may be beyond the reach of thought.

## 4. Quantum Measurement as Entanglement

As Bohr came to realize in the wake of his exchange with EPR [11,36], a quantum measurement has a subtler nature, which is parallel to that of EPR-type measurements even in the standard case of quantum measurement. In any quantum experiment, the *object* under investigation and the measuring instrument become entangled as a result of their interaction with each other. Technically, entanglement is a feature of the formalism of QM which reflects the feature of quantum phenomena defined by this interaction. For simplicity, however, I shall refer by entanglement to this situation as a whole and speak of the entanglement between the object and the instrument.

Further qualifications are necessary given the concept of quantum objects in the interpretation adopted here, as an idealization only applicable at the time of measurement. When one makes a prediction based on a given measurement, it can only concern a new possible quantum object, defined through the interaction between the ultimate constitution of the reality responsible for quantum phenomena and a new measuring instrument used and not an object that we measure in order to make the prediction. As explained in the preceding section, however, in QM or low-energy QFT, although not in high-energy QFT, assuming that our predictions concern the same quantum object as registered by the initial measurement is a permissible idealization. In any event, any rigorous statement concerning an entanglement can only refer to observable events, with which the idealization of a quantum object is, again, associated, while one cannot ultimately speak of the interaction between quantum objects between experiments. One can only speak of two quantum objects associated with the measurements performed initially and then two quantum objects associated with two measurements performed subsequently. Quantum entanglement can be defined in terms of such measurements, the outcomes of which QM properly predicts. The EPR experiment as such is beyond my scope. It suffices to say here that the entanglement between two quantum objects, *S*_1_ and *S*_2_, forming an EPR pair (*S*_1_, *S*_2_), allows one by means of a measurement performed on *S*_1_ to make predictions, with probability one, concerning *S*_2_. In the present view, *S*_2_ is only defined once the corresponding measurement is performed, but not when the prediction concerning it is made, which makes it even more difficult and rigorously impossible to speak of any independent properties of *S*_2,_ however predicted, because there is no *S*_2_, in the first place, until it is measured. There is only the independent, RWR-type, reality, ultimately responsible for the existence of *S*_2_, when it is measured, and secondly, even then *S*_2_ is still an RWR-type entity, which means that no physical properties can be attributed to it as such. These properties could only be attributed to the instrument used. As predictions at a distance, these predictions may be called “quantum-nonlocal” [7]. They do not, however, entail any instantaneous transmission of physical influences between such events, “a spooky action at a distance” [*spukhafte Fernwirkung*], famously invoked by Einstein [37] (p. 155). As such, they may be called Einstein-local. Einstein-locality would prohibit such an action, as would relativity, although the concept of Einstein-locality, or the locality principle, which implies that physical systems can only be physically influenced by their immediate environment, is independent of relativity.

The interaction between the object and the measuring instrument leading to their entanglement is not a measurement in the sense of *giving rise* to an observable quantity: this interaction occurs before the measurement takes place or rather before the outcome of this interaction is registered as a quantum phenomenon. This interaction is part of a quantum measurement, as defined here, which establishes a quantum phenomenon manifested in a measuring instrument or the data found in it, to which a measurement in the sense of measuring a physical property can then apply. Once performed, the measurement, say, that of the momentum (manifested only as a property of the instrument), disentangles the object and the instrument, with the observed outcome “irreversibly amplified” to the level of the classically observed stratum of the apparatus [2] (v. 2, p. 73). This outcome is defined by the quantum stratum of the apparatus after this interaction, rather than by the object. As Schrödinger explains in his cat-paradox paper, it is this disentangling that enables one to predict the probability that the momentum measurement at a given future moment in time will be within a certain range [21] (pp. 162–163). Alternatively, if the initial measurement was that of the position, one could predict the probability that the position measurement at a given future moment in time will locate the trace of the interaction between the object and the instrument within a certain area.

Quantum phenomena are never entangled. In the present view, again, not even quantum objects are entangled because they are idealizations only applicable at the time of measurement and thus always irreducibly associated with quantum phenomena. One could only say that two initial measurements associated with *S*_1_ and *S*_2_ lead to the situation in which possible future measurements can be handled by the mathematics of entangled states in the formalism of QM and expectation catalogs they enable. Accordingly, in the present view, only *quantum states*, ψ-functions, can be entangled, but there is something in the ultimate nature of reality responsible for quantum phenomena that requires this entanglement. If one assumes an independent existence of quantum objects between measurements, which is possible even in RWR-type interpretations, then one could say that they become entangled, although, if one adopts an RWR-type interpretation, the nature of the reality defining this entanglement is beyond representation or knowledge or even conception. ψ-functions never represent either the ultimate reality responsible for quantum phenomena or quantum phenomena and thus the outcomes of measurements. They do not represent these outcomes even if one adopts a realist view of ψ-functions as representing what happens between measurements because one needs Born’s rule added to the formalism to predict the probabilities of these outcomes, described by classical physics.

Now, according to Bohr’s remarkable observation, in effect describing the quantum measurement as an entanglement:

*After a preliminary measurement of the momentum of the diaphragm, we are in principle offered the choice, when an electron or photon has passed through the slit, either to repeat the momentum measurement or to control the position of the diaphragm and, thus, to make predictions pertaining to alternative subsequent observations. It may also be added that it obviously makes no difference, as regards observable effects obtainable by a definitive experimental arrangement, whether our plans of considering or handling the instruments are fixed beforehand or whether we prefer to postpone the completion of our planning until a later moment when the particle is already on its way from one instrument to another.*

[2] (v. 2, p. 57).

If, then, a measurement is always made after the object has left the location of the measurement, what does this measurement measure? How does it create the corresponding phenomenon? It “measures” the quantum state of the quantum stratum of the instrument, which interacted with the object in the past (however recently, but always in the past!), by amplifying this state to the classical level of the observation and, thus, by registering the corresponding state of the measuring instrument. This amplification leads to the phenomena in which the outcome of a measurement is registered. What will be registered is either the change in the momentum of certain observed parts of the apparatus or the position of one or another trace of this interaction, say, a spot on a silver screen, given that both can never be registered in the same arrangement, as reflected by the uncertainty relations. Such concepts as momentum or position can only rigorously apply at this classical level in the RWR view, adopted by Bohr by the time of this statement (1949). This point appears to have been missed either in commentaries on Bohr or by treatments of quantum measurement elsewhere. Subtle as it is, Schrödinger’s analysis of quantum measurement in his cat-paradox paper does not consider this point [21] (pp. 158–159). Neither does Ozawa in his analysis in [30], discussed earlier, an analysis that is expressly realist and implies that the measured quantity is attributed to the object at the time of measurement. Von Neumann’s analysis comes close, but, while it is conceivable that von Neumann realized this point, he did not comment on it, and some of his statements appear to attribute the measured quantity to the object at the time of measurement [16] (pp. 355–356). On the other hand, this aspect of quantum measurement supports the point, made by von Neumann and others, that an instantly repeated measurement will give the same result as the initial measurement [16] (pp. 214–215), [21] (pp. 158–159).

The situation is consistent with the present interpretation, according to which a quantum object is an idealization only applicable at the time of measurement, insofar as one refers by measurement, as Bohr clearly does, to the overall process in question, leading to the emergence of an observed phenomenon (in Bohr’s sense). The interaction is between the quantum object and the quantum stratum of the instrument and the amplification of the resulting quantum state of this stratum (after this interaction and hence no longer in the presence of the quantum object) to the classical level of the observed stratum of the instrument, in which stratum the outcome is registered. Even though the quantum object is no longer there, or even no longer assumed to exist, or be a viable idealization, when the corresponding phenomenon is established, one might still see this quantum object as, by its interaction with the quantum stratum of this instrument, responsible for the effect observed. It follows that, in the present view, a quantum object, as an idealization applicable at the time of a measurement, refers to, *idealizes*, something that physically existed in the past of an observed quantum event. An observed effect could also be that of the measurement of the charge, mass, or spin of an electron. These quantities will be the same for all electrons and will define them as electrons, in the present view at the time of measurement. Such quantities as the position, velocity, momentum, or energy registered in a measurement will be different. One might assume that, say, because of the exchange of momenta between the object and the instrument, the momentum of the object will correspond to the difference between two momentum measurements of the instrument before and after the interaction with the object. Physically, however, one never measures that momentum, given that the object has already left the location of the instrument and that one could have performed instead the position measurement after it did. In any event, one can ascertain, *regardless of an interpretation*: (a) that one can perform either of the two complementary measurements concerning the state of the quantum stratum of the instrument, with the outcome amplified to the classical level of the observable part of this instrument, and correlatively (b) the quantum-nonlocal nature of quantum predictions, because by changing one’s decision which measurement to perform, one can make two alternative predictions concerning distant future events, to which one is not physically connected at the time of either measurement.

Thus, using the measurement of the state of the apparatus, one can predict, at a distance, by means of a ψ-function (cum Born’s rule), a possible outcome of a future measurement of either variable, without “in any way disturbing the system,” just as in any EPR-type experiment [36] (p. 138). It is true that there was an interaction between the object and the instrument before that measurement. But this is also the case for the two objects of the EPR pair, which have been in an interaction, entangling them. In a standard measurement, the probability of such a prediction will not be equal to one, as it would be in the case of the EPR-type experiments, which possibility, however, requires qualifications, discussed below. Besides, as Bohr realized, with some simple additional arrangements one can, at least in principle, reproduce the EPR case in considering the standard quantum measurement [2] (v. 2, p. 60), [15] (pp. 101–103).

It *might seem* that, in either the standard or the EPR case, because either of the two complementary quantities could be predicted at a distance for one quantum object by an alternative measurement on another quantum object, the first object at a distance can be assigned both quantities, as, in EPR’s language, “elements or reality” “without in any way disturbing” it [36] (p. 138). This was, essentially, EPR’s argument, although the possibility of predicting either quantity with probability one in the EPR experiment strengthened their case. EPR also argued that the only alternative, in the case of the EPR experiments, would be that QM is Einstein-nonlocal, because a measurement on one object would alternatively define the state of reality at a distance [36] (p. 141). As, however, should be apparent from the comments just given and as discussed in detail by the present author elsewhere [7] (pp. 1847–1855), this is not necessarily the case, because, in any *actual* experiment, only one of these quantities could be predicted. There is no experiment that would allow one to physically realize the prediction of both quantities for the same object. At the same time, there is no need to assume that our predictions are Einstein-nonlocal by virtue of determining the quantity in question at a distance, because, even if one can predict this quantity with probability one, one could still measure the complementary quantity and thus establish, in EPR’s language, a different element of reality from the one predicted [36] (p. 138). A measurement performed on one quantum object cannot be claimed to define an element of reality pertaining to another, spatially separated, quantum object, by means of a prediction, even with the probability one, the situation discussed in detail under the heading of quantum causality in [7]. Only a measurement on this second object can do so. Accordingly, there is no rigorous basis for assuming that the latter prediction established the reality it predicts. In classical physics, these limitations do not apply because we can always measure and predict and simultaneously verify our predictions for conjugate variables, such that of position and that of momentum. The *conjugate variables* of classical physics are *not the same as complementary variables* of quantum physics.

The outline of the EPR experiment just given is only a sketch that requires a proper argument, given by thus author in [7] (pp. 1847–1855). The main point at the moment is that, even in the standard quantum measurement one must always consider not only the object under investigation as in classical physics (where one can disregard the role of measuring instruments) but a composite entangled quantum system, consisting of the object and the quantum stratum of the instrument [21] (p. 167), [20,38]. In each EPR-type experiment, at the last stage of the experiment, when one of the two possible EPR predictions is made, one deals with two combined systems, each consisting of an object and an instrument, the first associated with an actual (already performed) measurement and the other with a possible future measurement, concerning which one makes a prediction, and thus with four systems in total.

## 5. “Perhaps the Biggest Change of All the Big Changes in Physics”: Quantum Measurement, Quantum Objects, and Elementary Particles in QFT

This section extends the preceding analysis to high-energy quantum regimes and QFT, which also enables this article to address the problems of quantum field and elementary particles. Both have been and remain problems to which nothing more that fragments of possible solutions could be offered, as testified to by the persistent title, “What is an elementary particle?” used by, among others, Heisenberg [39] (pp. 71–88) and S. Weinberg [40]. Because I shall primarily deal with elementary particles, by particles I shall, unless qualified, refer to elementary particles. Of course, a “particle” is a problem, too, a problem that underlies that of an elementary particle and has been around for much longer than that of elementary particles.

It is not my aim here to do more than consider this problem as a problem. In contrast, however, to most approaches to this problem, which are realist in nature, this article offers a nonrealist one, based on the strong RWR view. As a result, my engagement with literature on the subject will be more limited than one might prefer. However, the sources cited here, such as [41,42,43,44], contain extensive bibliographies of both physical and philosophical literature. Among the standard technical textbooks are [45,46,47]. Most works on QFT in physics and the philosophy of physics, too, adopt and primarily address realist views. An extensive philosophical treatment of the concept of a particle is offered by [48]. The question of virtual particles is beyond my scope here. It was considered from the present perspective in a previous article by this author [9]. A compelling realist perspective on the subject and an effective critique of arguments against the existence of virtual particles was offered in [41,42].

The strong RWR view and the corresponding interpretations of QFT imply that the question “What is an elementary particle?” has ultimately no answer, at least as things stand now, insofar as this view allows for no specifiable concept of elementary particles or quantum objects in general, apart from in terms of their effects on measuring instruments, effects that are rigorously specifiable by means of classical physics. It need not follow that the ultimate nature of (RWR-type) reality responsible for quantum phenomena is constant or uniform. While each time unknowable or, in the strong RWR view, unthinkable, this reality is assumed to be each time different in its ultimate character as well as in its manifested effects, making each quantum phenomenon or event, as such an effect, unique in turn, as well as discrete in relation to any other quantum phenomena. Indeed, in contrast to low-energy quantum regimes (QM or QFT), in high-energy quantum regimes an investigation of a particular type of elementary particle unavoidably involves not only other particles of the same type, say, electrons, but also *other types* of particles, such as, in QED, positrons, photons, or electron-positron pairs, that is, dealing with the corresponding effects, even in the same experiment. By the same token, it becomes meaningless to speak of the same electron detected in any two successive measurements. While, in the present view, assuming the identity of two successively detected quantum objects is only a statistically permissible idealization even in low-energy quantum regimes, this assumption is no longer possible in high-energy quantum regimes. This situation further justified and gives additional significance to the tripartite view or idealization of physical reality adopted in this article: (1) the ultimate constitution of physical reality, as RWR-type reality, responsible for quantum phenomena; (2) quantum objects, including elementary particles, defined by measurement as RWR-type entities, and (3) quantum phenomena, also defined by measurement but represented classically. The identity of particles of the same type is strictly maintained, as it is in the case in QM or low-energy QFT. While applicable and helpful even in QM or in low-energy QFT, the concept of a quantum field introduced here is designed to handle these new types of effects observed in high-energy quantum regimes. Rather than, as is more common, as a quantum object, a quantum field is defined here as a form of the independent RWR-type reality responsible for quantum phenomena and quantum objects, such as elementary particles, at the time of measurement.

Low-energy quantum regimes permit and most interpretations of quantum phenomena and QM (or QFT in low-energy regimes) adopt a conception of elementary particles, as quantum objects. The same conception is also applicable in, although not sufficient for, high-energy regimes, beginning with the circumstance that elementary particles of the same type, such as electrons or photons cannot be distinguished from each other, while these types themselves are rigorously distinguishable. Two electrons could be distinguished by changeable properties associated with them, such as their positions in space or time, momentums, energy, or the directions of spins but, in the RWR view, only as properties manifested in measuring instruments and only at the time of measurement. Such properties are subject to the uncertainty relations and complementarity. It is possible to locate, and in the present view, establish, by measurement two different electrons, as quantum objects, in separate regions in space. It is not possible to distinguish them from each other on the basis of their mass, charge, or spin. These quantities are not subject to the uncertainty relations or complementarity. As H. Weyl observed long ago, “the possibility that one of the identical twins Mike and Ike is in the quantum state E1 and the other in the quantum state E2 does not include two differentiable cases which are permuted on permuting Mike and Ike; it is impossible for either of these individuals to retain his identity so that one of them will always be able to say ‘I’m Mike’ and the other ‘I’m Ike.’ Even in principle one cannot demand an alibi of an electron!” [49] (p. 241). In RWR-type interpretations, properties defining electrons or other elementary particles within each type could only be *associated with* them (even if one assumes their independent existence between measurements, as opposed to only assuming them to exist at the time of measurement) by means of the corresponding effects observed in measuring instruments, rather than properties attributable to these objects themselves. A rare discussion of the “properties” of the electron along similar lines, even if without adopting the RWR view, is offered in [50]. It is possible, however, to maintain both the indistinguishability of particles of the same type and the strict distinguishability of the types themselves in RWR-type interpretations because both features can be consistently defined by the corresponding sets of effects manifested in measuring instruments.

This view is, thus, in accord with the assumption, defining RWR-type interpretations, that the *character* of elementary particles and their behavior, or of the reality thus idealized, is beyond representation or even conception, just as is the ultimate, RWR-type, character of the reality itself responsible for quantum phenomena. In the present interpretation, this reality is, again, assumed to exist independently, while elementary particles, as quantum objects, only idealize something that is assumed to exist in quantum measurements. An elementary particle of a given type, say, an electron, is specified by a discrete set of possible effects (the same for all electrons), observable in measuring instruments in the experiments associated with particles of this type. An elementary particle can thus only an idealization that is part of a composite system, consisting of this particle and a measuring instrument, system which has a registered effect upon the observable, classically describable, part of this instrument. The elementary character of a particle is defined by the fact that there is no experiment that allows one to associate the corresponding effects on measuring instruments with more elementary individual quantum objects. Once such an experiment becomes conceivable or is performed, the status of a quantum object as an elementary particle could be challenged or disproven, as it happened when hadrons and mesons were discovered to be composed of quarks and gluons. This composite nature will then manifest itself in a new set of effects observed in the corresponding experiments.

The present concept of an elementary particle, defined, as an idealization, in terms of such effects, does not imply that “elementary particles” are fundamental elementary constituents, “building blocks,” of nature. This assumption is impossible in RWR-type interpretations, as is any assumption concerning this constitution. Nor is applying the concepts of “elementary” or “constituents” ultimately possible either. Nature has no elementary particles, which are human idealizations, albeit made possible by our interaction with nature, assumed, in its ultimate constitution, to be a form of RWR; nor, by the same token, is it possible to apply to elementary particles any concept of a particle, any more than any other concept, such as wave or field, although, as will be explained presently, the concept of a quantum field could be defined otherwise, as a mode of RWR-type independent reality (rather than a quantum object, such as an elementary particle) beyond the reach of all specifiable concepts.

While most QFT conceptions of an elementary particle are transferred from QM to high-energy quantum regimes, they are insufficient in these regimes and need to be adjusted or supplemented by additional concepts, most commonly that of a quantum field. The present approach follows this pattern by defining the concept of quantum field in RWR terms. First, however, I shall explain why the concept of an elementary particle operative in QM is insufficient in high-energy regimes. This insufficiency arises in view of the following situation, not found in QM (or low-energy QFT, even though the latter introduces new features into quantum phenomena), to which the mathematical architecture of QFT responds. (The low-energy QFT is essential for explaining some quantum phenomena, such as the non-zero the energy of the vacuum, not explicable by QM.) In fact, with Dirac’s equation, it was this architecture, discovered first, that led to the discovery of this situation.

Speaking for the moment in classical-like terms, suppose that one arranges for an emission of an electron, at a given high energy, from a source and then performs a measurement at a certain distance from that source, say, by placing a photographic plate there. The probability or, if we repeat the experiment with the same initial conditions (defined by the state of the emitting device), the statistics of the outcomes would be properly predicted by QED, but what will be the outcome? The answer is not what our classical or even quantum-mechanical intuition would expect. This answer was a revolutionary discovery made by Dirac through his equation.

Let us consider first what happens if one deals with a classical object analogous to an electron and then if one considers a nonrelativistic QM electron in the same type of arrangement. I speak of a classical object analogous to an electron because the “game of small marbles” for electrons was finished well before QM. An electron, say, a Lorentz electron, of a small finite radius, would be torn apart by the force of its negative electricity. This led to treating the electron mathematically as a dimensionless point, without giving it any physical structure, while still assigning it measurable physical quantities, permanent (such as mass, charge, or spin) or variables (such as position, time, momentum, or energy). However, a point electron in quantum theory is, as an idealization, different from a point-like idealization in classical mechanics, where the body thus idealized could still be assumed to have spatial dimensions. Thus, one can take as an example of the classical situation a small ball that hits a metal plate. The place of the collision could be predicted (ideally) exactly by classical mechanics, and we can repeat the experiment with the same outcome on an identical or even the same object. Regardless of where we place the plate, we always find the same object. (It is assumed that the situation is shielded from outside interferences, which could, for example, deflect the ball before it reaches the plate).

If one considers an electron in the QM regime, it is, first of all, impossible, because of the uncertainty relations, to predict the place of collision exactly or with the degree (ideally unlimited) of approximation possible in classical physics. An (ideally) exact prediction of the position (or other variables) of a quantum object is possible, specifically in EPR-type experiments by means of a measurement performed on the other particle of the (EPR) pair considered. As discussed earlier, however, even a prediction with probability one is still a prediction and not a guarantee of the reality of what is predicted, because one can always perform a complementary measurement, thus disabling any possibility of verifying such a prediction and hence assigning the corresponding quantity. In addition, a single emitted electron could, in principle, be found anywhere in a given area or not found at all. Nor can an emission of an electron be guaranteed. There is a small but nonzero probability that such a collision will not be observed or that the observed trace is not that of the emitted electron. Finally, assuming that one observes the same electron in two successive measurements is still an idealization in the present interpretation, given that it defines any quantum object as an idealization applicable only at the time of measurement. This idealization is, however, permissible in low-energy quantum regimes.

Once one moves to high-energy quantum regimes, beginning with those governed by QED, the situation is, again, different, even radically different. In a subsequent measurement, one can find in the corresponding region, not only an electron or nothing, as in low-energy (QM) regimes, but also other particles: a positron, a photon, or an electron–positron pair. That is, in RWR-type interpretations, one can register the events or phenomena (observed in measuring instruments) that we associate with such entities. QED predicts which among such events can occur and with what probability or statistics, and just as QM, QED, in RWR-type interpretations, does so without representing or, in the strong RWR view, allowing one to conceive of how these events come about. The corresponding Hilbert-space formalism becomes more complex, in the case of Dirac’s equation making the wave function a four-component Hilbert-space vector, as opposed to a one-component or, if one considers spin, two-component Hilbert-space vector, as in quantum mechanics, keeping in mind that each component is infinite-dimensional. These four components represent the fact that Dirac’s equation

$$\left(\beta mc^{2}+\overset{3}{\underset{k=1}{\sum }}\alpha_{k}p_{k}c\right)\psi \left(x,t\right)=i\hbar \frac{\partial \psi \left(x,t\right)}{\partial t}$$

$$\alpha_{i}^{2}=\beta^{2}=I_{4}$$

(*I*_4_ is the identity matrix)

$$\alpha_{i\;}\beta +\beta \alpha_{i}=0$$

$$\alpha_{i}\alpha_{j}+\;\alpha_{j}\alpha_{i}=0$$

is an equation for both the (free) electron and the (free) positron, including their spins, and they can transform into each other or other particles, such as photons, in the corresponding high-energy processes, transformations that, in the RWR view, are only manifested in measuring instruments. By the same token, one can no longer speak of the same electron, positron, and so forth as detected in two successive measurements in low energy quantum regimes. In the currently standard versions of QFT, the wave functions are commonly replaced by operators (the procedure sometimes known, for historical reasons, as “second quantization”): to every point *x* a Hilbert-space operator acting on this space is associated, rather than a state-vector as in QM. As M. Kuhlman notes: “both in QM and QFT states and observables [operators] are equally important. However, to some extent their roles are switched. While states in QM can have a concrete spatio-temporal meaning in terms of probabilities for position measurements, in QFT states are abstract entities and it is the quantum field operators that seem to allow for a spatio-temporal interpretation” [43]. As he (rightly) qualifies, however, “since ‘quantum fields’ are operator valued, it is not clear in which sense QFT should be describing physical fields, i.e., as ascribing physical properties to points in space. In order to get determinate physical properties, or even just probabilities, one needs a quantum state. However, since quantum states as such are not spatio-temporally defined, it is questionable whether field values calculated with their help can still be viewed as local properties” [43]. While the present concept of a quantum field is physical, all calculated values are only probabilities and all local properties are only those of the observable parts of measuring instruments, just as they are in QM.

Once one moves to still higher energies governed by QFT, the panoply of possible outcomes becomes much greater. Correspondingly, the Hilbert spaces and operator algebras involved have still more complex structures, linked to the appropriate Lie groups and their representations, defining (when these representations are irreducible) different elementary particles. In the case of QED, we only have electrons, positrons, and photons, single or paired; in QFT, depending how high the energy is, one can literally find any known and possibly as yet unknown elementary particle or combination. It is as if instead of identifiable moving objects and motions of the type studied in classical physics, we encounter a continuous emergence and disappearance, creation and annihilation of particles, further complicated by the role of virtual particles, or again, something in nature which compels some (not everyone sees it as necessary) to introduce the latter concept. This is still a classical-like and thus metaphoric picture, which is ultimately inapplicable. But we have no other pictures: if one wants to convey anything that “happens” between experiments by means of a picture or phenomenal concepts, rather than only use mathematics to predict what the outcome of experiments, classical-like pictures are our only recourse. Although, like anything quantum, these transformations can only be handled probabilistically or statistically, they also have a complex ordering to them. In particular, in addition to various correlational patters akin to those found in low-energy (QM) regimes, they obey various symmetry principles, especially local symmetries. The latter have been central to QFT, not the least in leading to discoveries of new particles, such as quarks and gluons inside the nucleus, and then various types of them, eventually establishing the standard model of particle physics. Thus, QED is an abelian gauge theory with the symmetry group U(1) and has one gauge field, with the photon being the gauge boson. The standard model is a non-abelian gauge theory governed by the tensor product of three symmetry groups U(1)⊗SU(2)⊗SU(3) and broken symmetries, and it has twelve gauge bosons: the photon, three weak bosons, and eight gluons.

The concepts (there have been quite a few) of a relativistic quantum field respond to the situation here outlined. (Most concepts of nonrelativistic quantum fields can be seen as limit cases of relativistic ones and will be put aside here, and, unless qualified, quantum fields will henceforth refer to relativistic quantum fields.) These concepts were initially developed as forms of quantization of the electromagnetic field, again, necessary even in low energy quantum regimes. The character and even the very possibility of such concepts, especially as physical concepts, is a subject of seemingly interminable debates, just as and often correlatively is the concept of elementary particles. While there is a strong general sense concerning the mathematics involved (although the range of specific mathematical tools offers one several choices) and while there is a large consensus that a viable physical concept of a quantum field is necessary, most of the proposals concerning such concepts are realist. This assessment is confirmed by Kuhlmann’s representative review [43]. By contrast, I suggest a nonrealist *physical* concept of quantum field defined by the strong RWR view, which is consistent with the mathematics of QFT and most currently available *mathematical* concepts of quantum field, such as those based on the Lagrangian formulation and canonical commutation or anticommutation relations for fields, for respectively bosonic and fermionic fields, analogous to those of QM, say, for bosonic field, ϕ and π:

$$\left[\phi \left(x,t\right),\pi \left(y,t\right)\right]=i\delta^{3}\left(x-y\right)$$

[ϕ(x,t),ϕ(y,t)]=[π(x,t),π(y,t)]=0

(Some of these mathematical concepts of quantum fields are associated with physical ones).

As understood here, a quantum field is *not a quantum object* but a particular *mode* of the RWR-type reality, which, as any such mode in the present view, is assumed to exist independently and is manifested only by its effects on measuring instruments, via quantum objects, such as elementary particles. A quantum field is independent of measurement, while quantum objects are always defined by measurements. These effects are more multiple than those observed, via the corresponding quantum objects, in low energy regimes. This multiplicity is defined by the fact that these effects correspond to elementary particles, to which a quantum field gives a rise and which can be of various types even in a single experiment, consisting of one or more successive measurements, with the first one performed on a given particle. The initial quantum object could also be a set of elementary particles of the same or different types, with a different such set, possibly consisting of entirely different types of particles, appearing in each new measurement. As a mode of the RWR-type reality assumed to exist independently, a quantum field is responsible for transforming effects associated with elementary particles created in the process at the time of measurement. These effects may be either invariant (as concerns a given particle type), such as those associated with mass, charge, or spin, or variable, such as those associated with position, momentum, or energy. As concerns this association, always via elementary particles, as quantum objects, there is no difference from low energy regimes; the difference is in what kind of effects are observed. These effects have a kind of multiplicity in high-energy regimes, not found in the case of effects observed in low-energy regimes. The multiplicities of types of elementary particles become progressively greater in higher-energy regimes. Hence, the concept of a quantum field just defined brings together the irreducibly unthinkable, discovered by QM, and the irreducibly multiple, discovered by QFT. It may be useful to comment, by way of a contrast, on the concept of a classical field.

A classical field is represented, in a realist way, by a *continuous* (technically, differential) manifold with a set of scalar (a scalar field), vector (a vector field), or tensor (a tensor field) variables associated with each point and the rules for transforming these variables by means of differential functions, from point to point of this manifold. One can also define it as a fiber bundle over a manifold with a connection. The concept of a fiber bundle is used in QFT, where it is associated with local gauge symmetry, in the RWR view, without representing, any more than any part of the mathematical formalism of QFT, any quantum physical process but only being part of the probabilistically or statistically predictive machinery of QFT. In classical physics or relativity, the variables in question map measurable quantities associated with the field, thus providing a field ontology, which also allows for (ideally) exact predictions concerning future events associated with this field via measurable field quantities. In quantum physics, in RWR-type interpretations, this type of ontology is impossible. One deals with a discrete manifold of phenomena and sets of quantities associated with each phenomenon, and hence, with a discrete manifold of quantities, without assuming any continuous process that would connect them. As does QM, QFT (in any regime) relates, in terms of probabilistic or statistical predictions, the continuous, technically, differential, mathematics to the discontinuous configurations of the observed phenomena and data.

As discussed in Section 2, in considering two successive measurements, which register different outcomes, it is humanly natural to assume that something “happened” or that there was a “change” in the physical reality responsible for these events between them. However, in any interpretation, one cannot give this happening a determined location in space and time, and in RWR-type interpretations, there is nothing we can say or even think about the character of this change, including as a “happening” or “change,” apart from its effects, the structures of which are more multiple in high-energy regimes than in low-energy regimes. Nor can one assume (again, in any interpretation), as one can, at least ideally, in low-energy regimes, that we observe the same quantum objects in two successive measurements. For example, it is no longer possible to think of a single electron (or for that matter a single proton) in the hydrogen atom, as the same electron detected by (and in RWR-type interpretations, defined) by different measurements. Each measurement is assumed to detect a different electron; if one makes a measurement between two measurements each of which detects an electron, this measurement can detect a positron or a photon, or an electron-positron pair. One could also speak of quantum fields in the sense defined here in QM (or in low-energy QFT), but in a reduced form that preserves the particle identities, as representatives of a given particle type—each photon always remains the “same” photon (or disappears), each electron the “same” electron (or disappears), and so forth, in the present view, again, strictly as a statistically permissible idealization because each is only defined by a measurement. In high-energy regimes, particles transform into one another, within and beyond a given particle type, also within because an electron could re-appear in this process, that is, be detected by a measurement, after a positron appeared in it after the previous appearance of an electron.

In this understanding, speaking, as is common, of the quantum field of a particle, say, an electron, entails new complexities. Mathematically, the formalism of, say, QED, allows one to make predictions concerning the electron, which, mathematically, invited one to speak of the electron as a quantum field. Physically, however, this only means that the RWR-type reality defining the quantum field considered in a given experiment has strata that enables the corresponding measurements detecting electrons. It is not possible to separate these strata from those similarly associated with the possibility of detecting a positron or a photon in the same experiment (in the sense of being defined by the same initial measurement) because neither of these strata as such is detected in measurement. It is possible, however, to specify quantum fields as associated with the fundamental forces considered in QFT and the corresponding types of particles, field bosons, electromagnetic (photons), weak *W^+^*, *W^−^*, and *Z*, or strong (gluons).

These considerations reflect the fact that the present concept of a quantum field is a physical concept, which defines a quantum field as part of the independent reality ultimately responsible for quantum phenomena and not as a quantum object, always dependent of an experiment. This concept can, however, be associated with most currently standard versions of a mathematical concept of a quantum field, defined in terms of a predictive Hilbert space formalism with a particular vector and operator structure, enabling the proper probabilistic predictions of the QFT phenomena concerned. The operators enabling one to predict the probabilities for the “annihilation” of some particles and “creation” of others, that is, for the corresponding measurable quantities observed in measuring instruments, are called annihilation and creation operators or also lowering and raising operators, commonly designated as *â* and *â*^†^, each lowering or increasing a number of particles in a given state by one. In RWR-type interpretations, these operators do not represent any physical reality: they only enable one to calculate the probabilities or statistics of the outcomes of experiments, just as the wave functions do in quantum mechanics. Both, to return to Schrödinger’s language, provide expectation-catalogs for the outcomes of possible experiments. Those provided by QFT give probabilities or statistics of the appearance of quantities associated with other types of particles even in experiments initially defined by a particle of a given type. In QFT regimes, it is, again, meaningless to ever speak of a single electron even in the hydrogen atom.

The description just outlined provides an RWR-type interpretation of the view, held even in realist interpretations, that the application of creation operators to quantum states (in the mathematical sense of vectors in a Hilbert space) does not represent a physical process of particle creation out of nothing, which would entail a violation of conservation laws. There are various ways of handling this situation in realist interpretations, for example, in terms of collisions (e.g., [51] (p. 73)). In the present view, this procedure and thus creation and annihilation operators are just part of the mathematical machinery that allows us to predict the transition probability between two quantum events, which would be associated with different particles, in the present interpretation, defined strictly by the measurements defining these events. Any measurement performed before the second measurement could reveal a different particle or set of particles than that found either in the first or in the second measurement.

The concept of a quantum field here defined does not introduce any new mathematics. It is not designed to do so. This concept is part of the (strong RWR-type) interpretation of quantum phenomena and quantum theory, and thus of how mathematics works quantum theory, especially in the case of QFT as different from QM, given the concept of quantum measurement and the tripartite scheme of physical reality in this interpretation, now redefined by configuring the ultimate constitution of physical reality responsible for quantum phenomena as that of quantum fields. As such this interpretation confirms the fundamentally mathematical nature of quantum theory, more radically so than that of classical physics or relativity, both of which are equally mathematical-experimental sciences, with mathematics coming first in this conjunction in all three types of theories. This is because in quantum theory the mathematics functions as an abstract formalism divorced from any physical representation and yet enabling us to relate to reality, including in its unrepresentable or inconceivable aspects, in terms of probabilities of the outcomes of quantum experiments. Indeed, as noted, while especially important to this understanding in high-energy quantum regimes and QFT, the concept of a quantum field proposed here is applicable in all quantum regimes, to the point of compelling one to conclude that, in quantum theory, the reality without realism is the reality of quantum fields.

Beginning with Dirac’s equation, QED and then QFT became a far-reaching extension of the revolution ushered in by Bohr’s 1913 atomic theory and Heisenberg’s discovery of QM, which theories also initiated the RWR view of quantum phenomena. This was acutely realized by Bohr and Heisenberg in assessing Dirac’s discovery of his equation and antimatter. Bohr spoke of “Dirac’s ingenious quantum theory of the electron,” as “a most striking illustration of the power and fertility of the general quantum-mechanical way of description,” which was, at this point, defined by Bohr’s ultimate, RWR-type, interpretation [2] (v. 2, p. 63). This “most striking illustration,” however, never convinced Einstein (whose debate with Bohr occasioned this statement) in the power and fertility of this method, nor many others who resisted this method, such as Schrödinger or Bell. Nevertheless, QED has not only amply demonstrated this power and fertility but is also the best confirmed physical theory ever. Heisenberg, who made major contributions to QFT, including that of nuclear forces, saw Dirac’s discovery as even more significant, even “as perhaps the biggest change of all the big changes in physics of our [twentieth] century. It was a discovery of utmost importance because it changed our whole picture of matter. … It was one of the most spectacular consequences of Dirac’s discovery that the old concept of the elementary particle collapsed completely” [39] (pp. 31–33). However, it also brought with it new ways of thinking about elementary particles and quantum fields, thus, at least in the present view, further confirming “the power and fertility of the general quantum-mechanical way of description,” a way that, as I have argued here, following Bohr, does not *describe* the ultimate constitution of nature responsible for quantum phenomena. It only describes how we interact with nature by means of our experimental technology and the mathematics of quantum theory, QM and QFT, enabling us to predict probabilistically the outcome of quantum experiments.

The question, then, becomes whether nature will allow us to do more, or, as there is no other way for us to do physics, whether we will be able to do more by means of our interactions with nature, which has no particles and makes no predictions. Einstein’s, or Bell’s, view was that we should. Bohr’s or at least the present (perhaps more cautious) view is that they *might not*, which, however, is not the same as claiming that they never will.

## Data Availability

Not applicable.
